# Geophysical investigations unravel the vestiges of ancient meandering channels and their dynamics in tidal landscapes

**DOI:** 10.1038/s41598-018-20061-5

**Published:** 2018-01-26

**Authors:** Jacopo Boaga, Massimiliano Ghinassi, Andrea D’Alpaos, G. P. Deidda, G. Rodriguez, Giorgio Cassiani

**Affiliations:** 1 Dipartimento di Geoscienze, Università di Padova, Padua, Italy; 2 Dipartimento di Ingegneria Civile, Ambientale e Architettura, Università di Cagliari, Cagliari, Italy; 3 Dipartimento di Matematica e Informatica, Università di Cagliari, Cagliari, Italy

## Abstract

Whether or not one can detect relict signatures of the past imprinted in current landscapes is a question of the utmost theoretical and practical relevance for meandering tidal channels, owing to their influence on the morphodynamic evolution of tidal landscapes, a critically fragile environment, especially in face of expected climatic changes. Unravelling the sedimentary patterns of ancient channels is an expensive process that usually requires high resolution sediment coring. Here we use a novel inversion process of multi-frequency electromagnetic measurements to reveal the signature and characterize the dynamics of a salt-marsh paleo-meander in the Venice Lagoon. We show that the ancient meander migrated laterally while vertically aggrading, developing a peculiar bar geometry which is less common in analogous fluvial meanders. The observed point-bar dynamics and the associated architectural geometry are consistent with remote sensing and borehole data and contrast with current assessments of tidal meander morphodynamics mediated from classical fluvial theories. In addition, the proposed technique, rapid and non-invasive, bears important consequences for detecting buried stratal geometries and reconstructing the spatial distribution of ancient sedimentary bodies, providing quantitative data for the description of landscape evolution in time.

## Introduction

Branching and meandering tidal channel networks cut through salt-marsh landscapes and drive the exchange of water, nutrients and sediments within these landscapes. Providing preferential pathways for marsh flooding and drainage during the tidal cycle, tidal channels exert a primary control on the ecogeomorphological evolution of salt-marsh systems e.g.^1,2^. The dynamics of salt-marsh platforms and of their channel networks are tightly intertwined e.g.^3–6^ while the channels drain and feed the marsh during the tidal cycle, governing water and sediment fluxes over the platform, the elevation of the marsh in the tidal frame, together with its extension, controls channel growth, maintenance, size and evolution^7–11^. Despite their importance in landscape evolution, tidal networks have received less attention than fluvial ones^12^ particularly in terms of the main morphometric characteristics of tidal meanders that are commonly studied following theories developed for their fluvial counterparts^13–17^. The internal structure of tidal point bars has received even less attention, attempts to address such an issue being indeed mainly based on similarities with fluvial meanders e.g.^18,19^. Improving current knowledge of tidal meander dynamics, and of the related sedimentary products, becomes an essential step to understand and predict tidal landscape morphodynamic evolution. Bar expansion, obstructed channels, outer–bank erosion and relocation of past meander bends can explain fining or coarsening-upward vertical grain-size trends, erosional surfaces and current salt-marsh topography e.g.^20^. The reconstruction of paleo-meander patterns can explain these morphological changes, and provide a significant contribute to reconstruct the depositional dynamics of paleochannels^21^.

The scientific issue above calls for solid evidence to develop detailed depositional models for tidal meander bends and related sedimentary products.

Several remote sensing techniques have widely been applied to map meander evolution in both tidal^15,22^ and fluvial^23–25^ landscapes. In this framework even a simple time-lapse aerial photography database, can supply relevant information on meander migration, especially if it spans a long time period^23,25,26^. Nevertheless, remote sensing techniques must be confirmed by ground-truthing, requiring detailed datasets of invasive, ancillary subsoil investigations^27,28^. A step forward is clearly needed.

Spatially extensive geophysical surveys give a fundamental contribution towards the characterization of the shallow subsoil and its heterogeneities, that can be related to geomorphological evolutions and different sedimentation processes in tidal landscapes^29,30^. A possible approach, both fast and accurate, is provided by electromagnetic techniques that allow one to perform contactless measurements^31^.

Here we present an innovative method to unravel the footprints of ancient meander bends in salt-marsh platforms and we test this new approach by recovering sedimentary cores that demonstrate that imaged sedimentary features are consistent with both observed deposits and recently established depositional models^20^. We show that the use a multi-frequency conductivity meter and the application of an innovative inversion technique of multi-frequency electromagnetic data (see Methods) allow one to unravel the geometry of a buried tidal point bar and related channel, which cut through a salt-marsh platform in the Venice lagoon (Italy), where saline soils are extremely conductive, thus representing a very challenging environment (in terms of discerning electrical conductivity spatial variations). Note that the presence of this paleo-meander is clearly shown by historical aerial photographs, whereas sediment accumulation over the past few decades has totally smoothed the marsh topography, covering direct morphological evidence of this channel. We also show how the three-dimensional geophysical data add critical information to the understanding of sedimentary processes in tidal landscapes. The integration between geophysical data, remote sensing images, and ancillary sedimentological investigations shows that salt-marsh aggradation plays a key role in developing geometries of tidal point bars. We present the peculiar morphodynamic behavior of tidal meanders, unusual for their fluvial counterparts, that is functionally intertwined to the evolution of the adjacent platform. We provide evidence that a multi-frequency electro-magnetic data inversion technique is a viable tool that can be used to study tidal meander evolution as well as the evolution of similar depositional environments. In addition, our findings challenge the possibility of applying fluvial meander theories to the study of their tidal fellows.

## The study site

**Figure 1:**
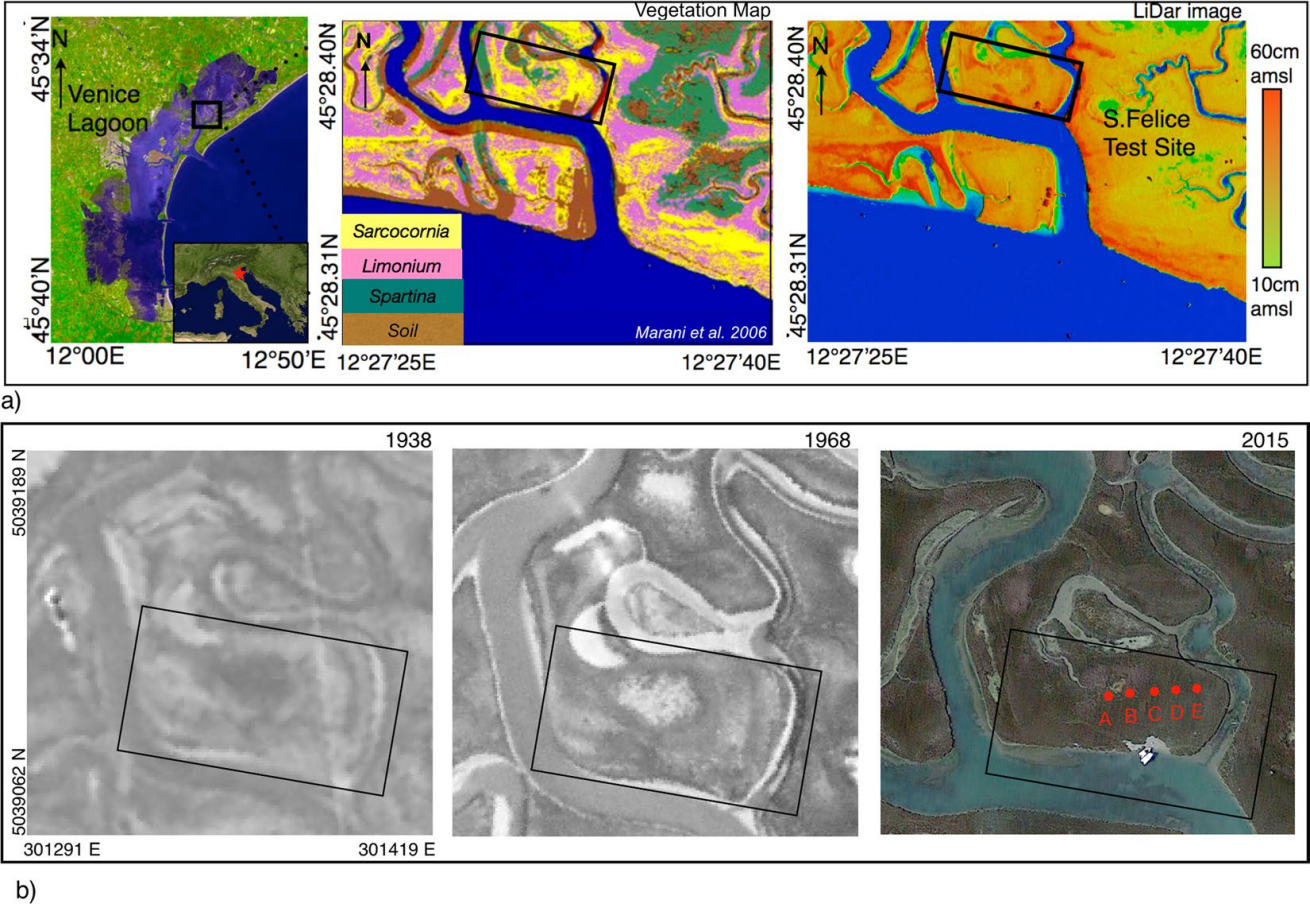
(**a**) Map of the test site in the S. Felice salt marsh, Venice Lagoon, N-E Italy (from Google Earth, modified with Iwork 5.3 suite https://www.apple.com/iwork/). The panel on the middle shows the vegetation map of the San Felice Marsh (from Marani *et al*. 2006^53^ -free of use under the Creative Commons Attribution license- modified, https://www.plos.org/license, modified with Iwork 5.3 suite https://www.apple.com/iwork/). The panel on the right shows a LiDar image of the study site with color-coded topographic elevations (from Boaga *et al*.^30^ - free of use under the Creative Commons Attribution license- modified, http://onlinelibrary.wiley.com/journal/10.1002/%28ISSN%291944–8007/homepage/FundedAccess.html, modified with Iwork 5.3 suite -https://www.apple.com/iwork/-). The black box shows the abandoned meander area, investigated in this study. As it can be seen in right panel the abandoned meander inner part presents a slight topographic height. This relative height in is confirmed by the vegetation map, which presents Sarcocornia vegetation, respect to the Limonium vegetation that usually characterize lower lands. (**b**) Historical aerial images (from the 1938 and 1968 flights, respectively (provided by the Magistrato alle Acque — Consorzio Venezia Nuova -Venice). Originals are freely available only at http://mapserver.iuav.it/website/foto_aeree/, while a brief description of sources and images are given at http://www.iuav.it/SISTEMA-DE/Laboratori1/patrimonio/aerofotogr/index.htm. Current satellite image of the S. Felice salt marsh (from Google Earth). The red dots A –E show the locations of two test boreholes.

The Venice Lagoon, which formed over the last 7500 years covering alluvial Late Pleistocene deposits^32^, is the largest Mediterranean brackish water body, with an area of about 550 km^2^ (Fig. 1a). The Venice Lagoon is subjected to a semidiurnal tidal regime, with an average tidal range of about 1.0 m and peak tidal amplitudes of about 0.75 m around Mean Sea Level (MSL). It is connected to the Adriatic Sea through three inlets: Lido, Malamocco, and Chioggia. The study site is located in the Northern part of the lagoon (Fig. 1a), which hosts the best naturally preserved network of meandering channels. The evolution of this network has been characterized by a few stream piracies, that caused the abandonment of channel reaches and their consequent burying under salt-marsh deposits.

We selected a study area where historical aerial photographs (e.g. the 1968 photo) show remnants of old tidal meanders (Fig. 1b), which used to drain the marsh platform in the past. Moreover the study area is close to a recently investigated site, where extended core drilling showed that the morphodynamic evolution of a tidal meander was affected by the aggradation of the surrounding salt-marsh platform^20^. Specifically, as it emerges from the 1968 aerial photo, the surveyed study site is characterized by arcuate lineations (hereafter “scroll-like”features) resembling different growth stages of a meander bend that expanded toward SE (Fig. 1b). At present, the site is covered by a dense halophytic vegetation (Fig. 1b), whose current zonation (Fig. 1a second panel), together with the small differences in marsh elevations (Fig. 1a third panel), suggest the presence of these paleo-meanders, which are not clearly visible when standing on the marsh platform. At this site we conducted a spatially dense, multi-frequency electromagnetic (EM) survey covering an area of approximately 70 m × 35 m.

## Results

**Figure 2:**
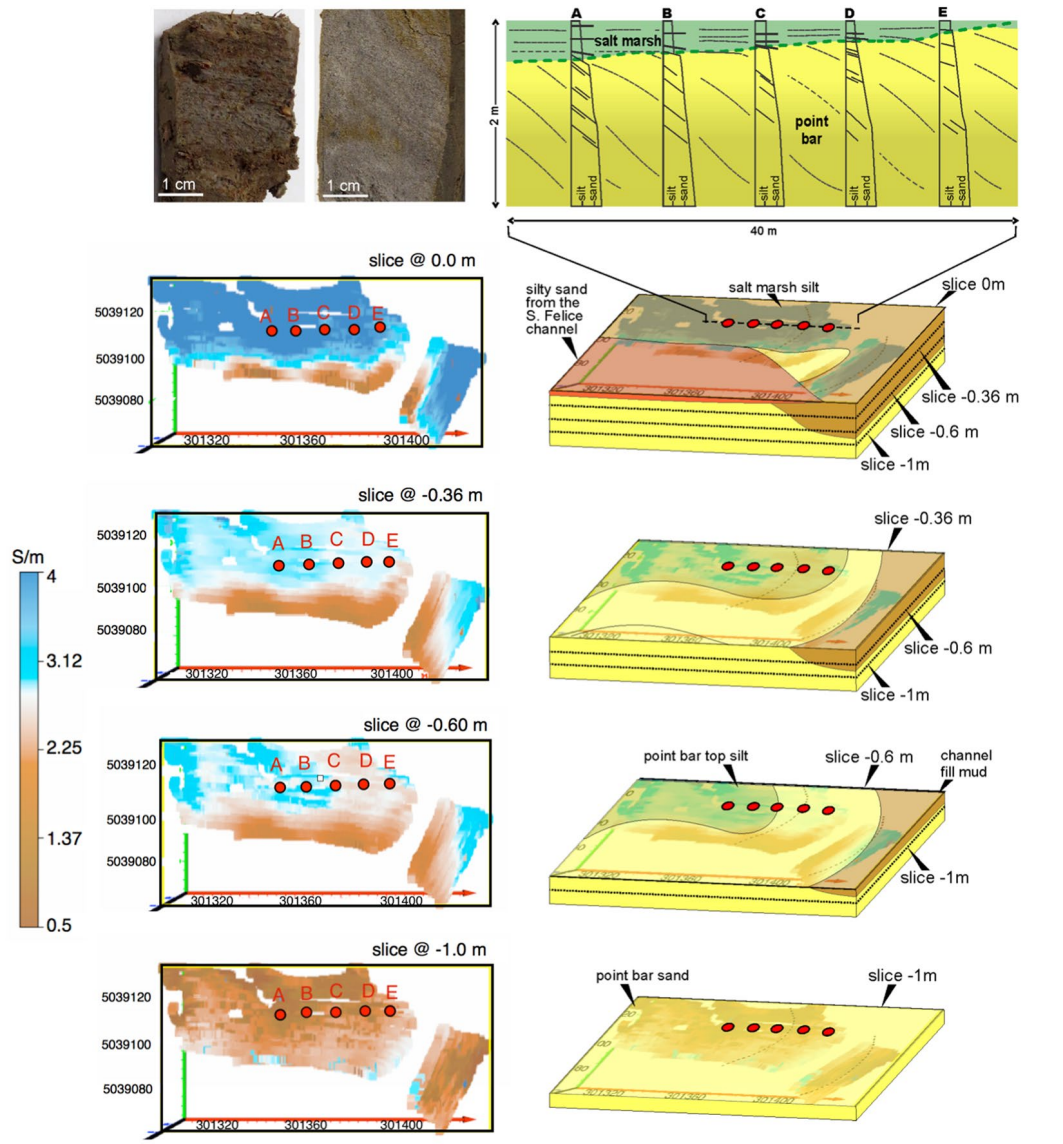
Electrical conductivity maps, at different depths, derived from electro-magnetic data inversion. Conductivity values are shown for the 5038 measurement points without interpolation (water channels and deep ponds could not be investigated). The 4 panels show the following layers: i) surface 0.0 m, ii) 0.36 m depth, iii) 0.60 m depth and iv) 1.0 m depth. Coordinates are expressed as meter in UTM zone 33T. A-E represent the locations of the boreholes. The maps are obtained from inverted field data using Surfer v.10.3.705 by Golden Software Inc.

The inversion of the acquired multi-frequency electromagnetic data allowed us to define the 3D stratal architecture of the site, that is shown in Fig. 2 where four depth slices are shown. Note that data are missing in correspondence of active, deep salt-marsh channels, where the acquisition could not be performed. In addition, all slices deeper than 1.0 m show essentially no spatial variation in electrical conductivity and a stable value around 2 S/m. In the region shallower than 1 m, both high- (>2 S/m, in blue) and low- (<2 S/m, in brown) conductivity regions are clearly marked. These analyses allow us to clearly discriminate a higher electrical conductivity domain (values > 3 S/m) from a lower conductivity domain. In the slice at 0.60 m depth, the distribution of the high-conductivity domain defines an ESE-WNW trending lobe, that increases in size at a depth of 0.36 m, and is prevalent at the ground surface (0.0 m). At 0.36 m depth, the Southern part of the study area, facing the deeper channels, is characterized by lower electrical conductivity values (brown areas), due to the presence of silty-sandy sediments spread from the main channel onto the marsh surface during flood events.

**Figure 3:**
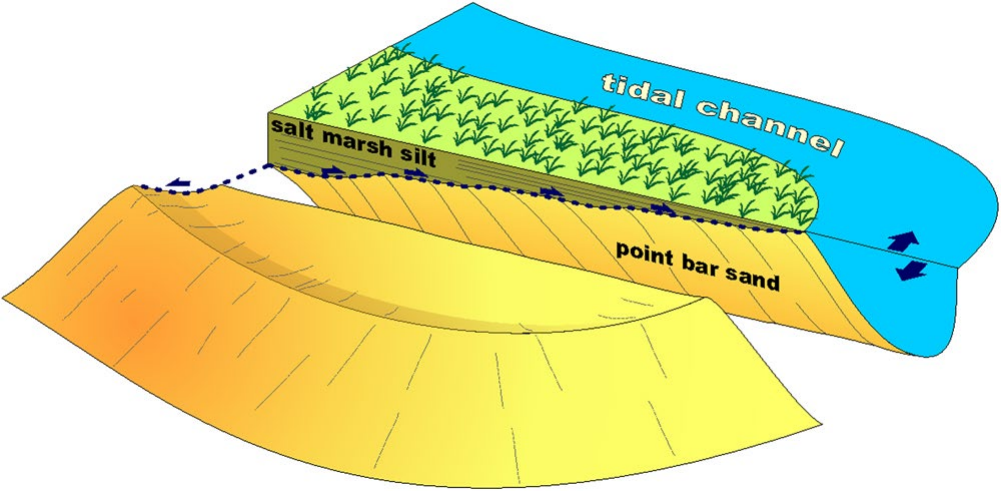
Conceptual model showing lateral migration of a tidal meander under aggradational. The blue dotted line shows the trajectory defined by the inner bank during bar growth. The drawing was prepared by M.G.

The distribution of the electrical conductivity values at different depths reflects the spatial orientation of scroll-like features that are visible from historical aerial photos (Fig. 1b). Drilling core data (Fig. 2) confirm the presence of the two different domains visualized by the EM survey, showing that the high- and low- conductivity domains are associated with salt-marsh silty-mud and point-bar sandy deposits, respectively. The spatial correlation between conductivity maps highlights that salt-marsh deposits reach their maximum thickness (~1.0 m) in the NNW sector of the surveyed area and that their basal surface is characterized by a “spoon-shaped” geometry with a SSE-NNW trending axis, as shown in Fig. 3.

## Discussion

Our study demonstrates the applicability and value of non-contact EM surveys in defining subsurface geometries even in electrically conductive environments such as salt-marsh systems, where the sensitivity of the measurement is stretched to the limit of detecting subtle spatial variations. Here the detection of subtle electrical conductivity differences, around the high values expected due to the prevailing presence of brackish waters, made the target particularly challenging. Only an algorithm capable of going beyond the low induction number approximation^33^ allowed us to draw the necessary conclusions in this environment. The results are - informative for the geometrical reconstruction of ancient tidal deposits, and thus allow us to detect the remnants of buried channels and cast light back in time on the past sedimentary processes. The integration between EM and sedimentological borehole data reveals that point-bar deposits associated with the studied paleomeander are now buried by salt-marsh silt ranging in thickness between 0.3 and 1.0 m. In particular, the higher electrical conductivity domain presenting values > 3 S/m (Fig. 2) can be related to the silty salt marsh deposits, while the lower conductivity values are representative of the more sandy point bar body (Tab.1 and Fig. 3). In these terms, the non-invasive electrical characterization of the buried meander structure suggests that the bar-top, salt-marsh deposits are characterized by a peculiar spoon-shape geometry, which is both validated by testing boreholes and consistent with recently proposed depositional models developed in similar bar deposits^20^, which, on the other hand, required recovering and sedimentological analysis of 150 cores. The subtle changes of electrical properties detected through the proposed model, are, therefore, representative of different sediment depositions, as confirmed by the drilled boreholes. The deposition of fine-grained sediments on point bar top reflects the morphodynamic evolution of channel bends, being they fluvial or tidal, and follows lateral migration/shifting of the main channel. Once salt-marsh deposition is triggered on the bar top, its aggradation is governed by the mutual role of the rate of relative sea level rise and of the deposition rate of organic and inorganic sediments^34^. At equilibrium conditions, as in the San Felice area^35–37^, the salt-marsh surface aggrades vertically keeping pace with the rate of relative sea-level rise (estimated to be about 3.0 mm/yr^31^). This implies that lateral migration of meanders cutting through the salt marsh occurs together with the progressive rise of bar top. We argue here that the effects of channel lateral migration under aggradational conditions are reflected by the geometry of the surface bounding the base of the salt-marsh deposits (Fig. 3). The lateral shift of the inner bank defines, through time, a concave-up rising trajectory (that we have referred to as “spoon shaped”) that depicts the basal surface of salt-marsh deposits. Along a cross section cut along the bar axis (Fig. 3), this concave-upward surface defines a rising trajectory, the steepness of which is controlled by the ratio between the rate of vertical aggradation and that of lateral migration. Meanders developed in the San Felice area show an average migration rate of about 10 cm/yr 14^38,39^, consistent with the few data available for other salt-marsh settings e.g.^22,26^. Aggradation rates of a few millimeters per year are therefore large enough to produce a detectable geometrical expression. To our knowledge, analogous processes and meander dynamics have never been documented in the fluvial realm. Indeed, similar features and behaviors can hardly be appreciable in the fluvial setting, where lateral migration rates can be more than one order of magnitude larger than those documented for salt-marsh meanders^20,22,26,38,39^, and only aggradation rates higher than 1.0 cm/yr are suggested to influence depositional dynamics^34^.

The San Felice area was characterized, during the past centuries, by an average salt marsh aggradation rate of 2.0–3.0 mm/yr^35,36^. The thickness distribution of salt marsh deposits above the point bar highlights that they thin from 1.0 to 0.3 m in the direction of bar expansion, suggesting that the channel migrated over a distance of about 40 m, between 400 and 120 yrs BP, if we consider an average accretion rate of about 2.5 mm/yr. The resulting migration rate of about 10 cm/yr is therefore consistent with that obtained for the salt-marsh meanders of the San Felice area^38,39^, for tidal-flat meanders of the Venice Lagoon^40^ and for salt-marsh channels in the San Francisco Bay^22^, and New Jersey^26^. The minimum thickness (0.3 m) of salt-marsh deposits indicates that since 120 yrs BP vertical aggradation started draping and flattening the pre-existing topography.

## Conclusions

A multi-frequency EM inversion approach was applied to detect relict signatures of a buried tidal meander in a salt marsh of the Northern Venice lagoon. The use of multi-frequency simultaneous acquisitions and of a robust inversion process, able to span the range of moderate induction numbers, provided detailed conductivity maps of the first subsoil even in such a challenging high-conductivity salty environment. The results of the EM survey were tested against data obtained from boreholes and remote images to reconstruct the sedimentary bodies developed during meander evolution. This is a notable achievement per se, providing a new tool for making new advance possible in different geoscience disciplines in saline environments. Data integration shows that vertical aggradation can influence the geometry and dynamics of sedimentary bodies generated during meander migration, leading to a progressive thickening of the point bar body during meander expansion, as recently pointed out in similar environment^14^. Changes in the thickness of salt marsh deposits accumulated on point bar top during meander evolution suggest that the channel migrated laterally with a rate of about 10 cm/yr, while vertically aggrading with a rate of about 2.5 mm/yr. The lateral migration rate is consistent with migration rates estimated for tidal-flat and salt-marsh channels in the Venice Lagoon^32,35^, San Francisco Bay^16^ and New Jersey^20^ wetlands. The emerging spoon-shaped geometry of the surface bounding the base of the salt-marsh deposits, which was observed in the Venice salt-marshes and has never been documented in fluvial cases, shed a new light in understanding tidal meander morphodynamics.

The use of fast, non-invasive techniques can therefore significantly help the evolutionary interpretation of the marsh sub-environment, even though the support of a limited number of ground punctual direct investigations, such as boreholes, is required.

## Methods

The field electromagnetic (EM) survey was conducted in November 2014. We collected the data using a Geophex GEM-2 conductivity-meter. The instrument has fixed transmitter-receiver coil separation of 1.66 m, operating with a multi-frequency acquisition in the bandwidth between 330 Hz to 48 kHz. The selection of frequencies depends on the sought depth of investigation and operates in the range of moderate induction number^40^. It must be emphasized that in salt marshes actual low induction number (LIN) conditions cannot be ensured, due to the highly electrically conductive environment. We used six frequencies (775 Hz, 1175 Hz, Hz, 9,825 Hz, 21,725 Hz, and 47,025 Hz) to span the range of induction numbers between 0.0918 and 0.715. Both quadrature (Q) and in-phase (I) components of the secondary magnetic field were recorded at roughly 5,000 measurement points across the survey area. The instrument was carried in the vertical-dipole configuration at the height of about 1.0 m above the ground. The conductivity meter was equipped with a differential GPS receiver that ensured sub-meter accuracy positioning for each measurement point.

Raw data were preliminary analyzed for detecting possible DC (static) shifts, outliers, and short wavelength noises, which usually adversely affect the quality of the inversions. The data show high sensitivity both responses in phase and quadrature vary over orders of magnitude in the selected frequency range. Note that this is peculiar to applications of multi-frequency EM instruments in presence of high electrical conductivities, as is the case in a salt-marsh environment. The adopted methodology is therefore very suitable for the problem at hand. However, the examination of the raw data reveal also unexpected features of the electromagnetic response. The in-phase (I) component of the three lowest frequencies turned out to be negative all over the area suggesting the presence of magnetic susceptible materials^41^ that were not expected. The quadrature (Q) response often presents a non-monotonic dependence with frequency. As debated in the literature^42^, this is usually not an issue for resistive soils, but can lead to serious non-uniqueness problems in the inversion process for highly conductive soils in the intermediate induction number range^33^. In this study we therefore used for inversion only the in-phase (I) component.

The filtered data were first inverted to produce a 1D electrical conductivity profile below each measurement point. Then, these 1D models were set side by side to build a pseudo-3D volume of the investigated area. The 1D inversions were performed using the regularized nonlinear inversion algorithm by Deidda *et al*.^43^. This approach, originally designed to invert the quadrature component measured at different heights, using a device operating at a fixed frequency, was here modified to handle multi-frequency in-phase component data^44^. The forward modeling was derived from Maxwell’s equations, introducing suitable simplifications based on the geometry of the instrumentation. When the axes of the coils are aligned vertically to the ground, the ratio of the secondary EM field *H*_*s*_ with respect to the primary EM field *H*_*p*_ can be expressed in terms of a Hankel transforms of order zero:

1 $$\frac{{H}_{S}}{{H}_{P}}={\int }_{0}^{\infty }{\lambda }^{2}{e}^{-2h\lambda }{R}_{0}(\lambda ){J}_{0}(r\lambda )d\lambda $$

where *h* is the height of the instrument above the ground, r the coil separation, *J*_0_ the Bessel function of order 0. The kernel *R*_0_ (*λ*) is a complex value function of the parameters that describe the layered subsurface properties, i.e. for the *k*-th layer: the electrical conductivity *σ*_*k*_, the magnetic permeability *μ*_*k*_ and the layer thickness *d*_*k*_.

The inversion originally proposed by Deidda *et al*.^43^ implements various methods for the automatic estimation of the regularization parameter. The inversion algorithm is designed to operate in a highly conductive setting, and is particularly fast compared to others^45^, since Deidda *et al*.^43^ propose the analytical computation of the Jacobian (sensitivity matrix), instead of approximating it by finite differences. Such a procedure makes the inversion more than ten times faster. In the inversion process we adopted a layered model consisting of 30 layers to a maximum depth of 3.5 m. To reproduce the negative in-phase data measured at this site, the relative magnetic permeability (*μ*_*r*_ = *μ*_*k*_/*μ*_0_, where *μ*_0_ is the magnetic permeability of the vacuum) was set *μ*_*r*_ = 1 at depths less than 0.5 m, *μ*_*r*_ = 1.14 between 0.5 m and 1 m, *μ*_*r*_ = 1.18 between 1 m and 3 m, and *μ*_*r*_ = 1.3 below 3 m. This increase in magnetic permeability with depth is compatible with the anoxic conditions present in the salt-marsh landscape^46,47^. Note that when the magnetic permeability for each layer is fixed, the conductivities *σ*_*k*_ are the only unknowns of the problem. The results emerging from the geophysical data have been tested against direct evidence from a few cores collected on site, as discussed in the main text^48–52^.
